# Heritability and genetic variance estimation of Osteosarcoma (OSA) in Irish Wolfhound, using deep pedigree information

**DOI:** 10.1186/s40575-021-00109-y

**Published:** 2021-10-09

**Authors:** Mehdi Momen, Nyah L. Kohler, Emily E. Binversie, Mariellen Dentino, Susannah J. Sample

**Affiliations:** 1 Department of Surgical Sciences, School of Veterinary Medicine, University of Wisconsin-Madison, 2015 Linden Drive, Madison, WI 53706 USA; 2 Irish Wolfhound Foundation, Litiz, PA 17543 USA

**Keywords:** Veterinary, Genetics, Irish wolfhound, Osteosarcoma, Dog, Canine, Heritability

## Abstract

**Background:**

Osteosarcoma (OSA) is a devastating disease that is common in the Irish Wolfhound breed. The aim of this study was to use a pedigree-based approach to determine the heritability of OSA in the Irish Wolfhound using data from a large publically available database.

**Results:**

The pedigree used for this study included 5110 pure-bred Irish Wolfhounds, including 332 dogs diagnosed with OSA and 360 control dogs; dogs were considered controls if they lived over 10 years of age and were not reported to have developed OSA. The estimated heritability of OSA in the Irish Wolfhound was 0.65.

**Conclusion:**

The results of this study indicate that OSA in the Irish Wolfhound is highly heritable, and support the need for future research investigating associated genetic mutations.

## Plain English summary

Osteosarcoma is a devastating condition that is prevalent in the Irish Wolfhound breed. In this study, our aim was to estimate heritability of osteosarcoma in the Irish Wolfhound breed. We undertook a pedigree-based analysis to estimate heritability of osteosarcoma in the Irish Wolfhound. The pedigree used included 5110 pure-bred Irish Wolfhounds, including 332 dogs diagnosed with osteosarcoma and 360 control dogs. We considered dogs to be controls if they were over 10 years of age and were not reported to have developed osteosarcoma. This study found the heritability estimate of osteosarcoma in the Irish Wolfhound to be 0.65. This score means that osteosarcoma in this breed is: 1) highly heritable and 2) a complex trait, which means that both environmental and genetic factors influence disease risk. Overall, our results provide support for further investigation into the genetic variants involved in the development of osteosarcoma in Irish Wolfhounds.

## Background

Osteosarcoma (OSA) is an aggressive bone cancer characterized by early metastasis and high mortality rates. Primary tumor removal and chemotherapy results in median survival times of 8–11 months [1, 2]. In dogs, the risk of OSA development is associated with breed [3]. The Irish Wolfhound breed has one of the highest incidences of OSA and the youngest age of onset [4]. The prevalence of OSA in the Irish Wolfhound is not well established, although it has been estimated that over 20% of the Irish Wolfhound breed dies from OSA [5].

OSA in dogs has proven a robust treatment model for OSA in children [6]. As a cancer, OSA in dogs is expected to be a complex genetic disease, as is the case with human OSA [7]. However, a subset of highly heritable cancers in humans and dogs can be highly influenced by single genetic variants. In humans, cancers with this type of inheritance pattern are often referred to as hereditary cancers, with risk largely attributed to a cancer predisposition gene [8]. To date, genomic information regarding the architecture of OSA in the Irish Wolfhound is suggestive that it is a highly heritable cancer. The only genome-wide association study evaluating OSA in dogs evaluated the genetic contribution to OSA in three breeds, including the Irish Wolfhound [9]. This study indicated that the genetic contribution towards OSA varies between breeds [9] and identified 4 candidate loci associated with OSA in the Irish Wolfhound. Meta-analysis of genome-wide association studies of OSA in dogs further indicated that for individual dogs, Mendelian-level polygenetic risk is present [10]. These studies formed a fundamental basis of understanding OSA in breeds with a high disease risk, but did not report an estimate of heritability. Further insight into Irish Wolfhound OSA can be seen through studies investigating the closely related Scottish Deerhound. In the Scottish Deerhound, OSA appears to be inherited in an autosomal dominant manner in certain families [11, 12], and a single genetic locus has been found to be associated with OSA in this breed on CFA34 [13].

Heritability of disease is important to define when comprehensively investigating the genetic architecture of a complex disease. Simply, heritability defines how much genetics influence a trait in a given population versus how much of disease risk is associated with environmental and epigenetic effects [14]. Thus, heritability estimates provide a qualitative measure of genetic influence, which can inform approaches to genetic investigation and, in some cases, inform selective breeding of animals known to possess a given trait. Furthermore, heritability estimates can inform whether pursuit of work to estimate genomic prediction is of value, as there is a direct relationship between heritability estimates and expected predictive test accuracy [15].

OSA in the Irish Wolfhound breed is devastating both on an individual level and as a breed. We utilized a large publicly available online Irish Wolfhound pedigree database, through which age and cause of death of thousands of pure-bred Irish Wolfhounds are available [16]. Given the high rate of OSA within the Irish Wolfhound breed [5], indications that OSA in some breeds of dog may be inherited with an autosomal dominant pattern [11, 12], and the availability of a large data set of Irish Wolfhounds, this study was designed to evaluate the heritability of OSA in the Irish Wolfhound and determine the mode of inheritance in Irish Wolfhounds.

## Results

### Pedigree and family structure

To evaluate pedigree structure for the Irish Wolfhound dog sample we used in this study, we extracted the summary statistics, as reported in Table 1. In total, 5110 dogs were included in the population-based pedigree. Of these, 2419 dogs (47%), showed some degree of inbreeding. A high ratio of inbred animals in a population indicates mating of more closely related individuals than the average mating relationship within a population and a low ratio shows mating of dogs with two apparently unrelated parents. The population investigated consisted of 1826 sires and 2666 dams; 4492 dogs had at least one progeny and 618 dogs had no offspring. There were 1108 founder dogs, defined as dogs that did not have parents recorded within the pedigree, and 4002 non-founders. The pedigree structure analysis showed that there were 575 full-sib families with an average size of 2.75 siblings per family. The average numerator relationship was 0.015 and the average number of discrete generation equivalents was 4.32 (Table 1).

**Table 1:** Structure and features of the Irish Wolfhound pedigree used

**Statistic**	**Number of dogs**
Individuals in total	5110
Total Inbreed dogs	2419
Sires in total:	1826
-Progeny	4002
Dams in total:	2666
-Progeny	4001
Individuals with progeny	4492
Individuals with no progeny	618
Founders:	1108
-Progeny	862
-Sire:	407
-Progeny	515
-Dam:	688
-Progeny	797
-With no progeny	13
Non-founders:	4002
-Sire:	1419
-Progeny	3487
-Dam:	1978
-Progeny	3204
-Only with known sire	1
-Only with known dam	0
-With known sire and dam	4001
Full-sib groups:	575
-Average family size (min-max)	2.726 (2–21)

### Pedigree based inbreeding coefficient

**Fig. 1:**
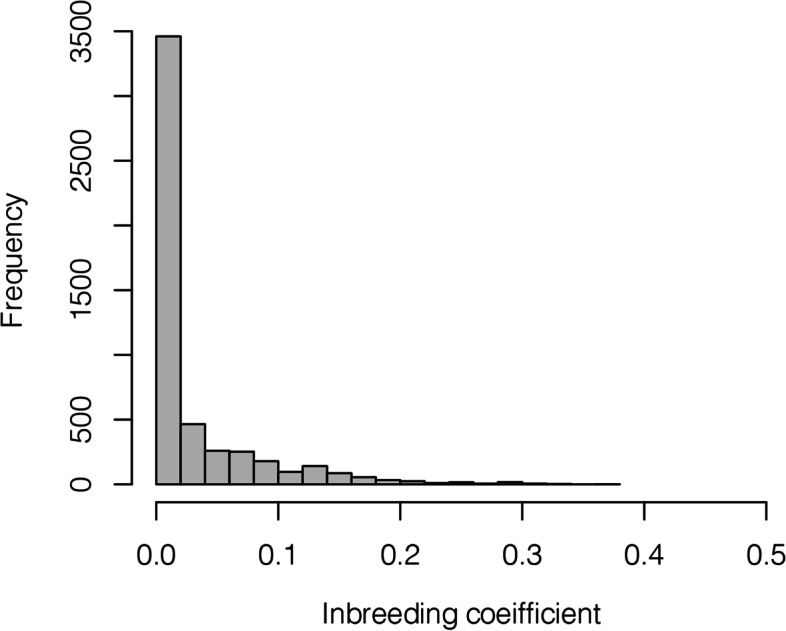
The histogram of inbreeding coefficients for Irish Wolfhound dogs in the pedigree, using diagonal element of the numerator relationship matrix

To evaluate the pedigree based inbreeding coefficient, we created numerator relationship linkage matrix. Figure 1 and Table 2 represent the distribution of inbreeding coefficients for the study population used. Inbreeding coefficients represent the probability of identity in the state of different pairs of genes, which means that two genes are of identical allelic type from a common ancestor. Therefore, a high inbreeding coefficient indicates an increase in the pairing of identical genes in the genome of an individual [17, 18].

**Table 2:** Distribution of inbreeding coefficients

Inbreeding coefficient range	Number of dogs	Percentage (%)
*F* = 0	2691	52.66
0 < *F* ≤ 0.05	1391	27.22
0.05 < *F* ≤ 0.1	534	10.45
0.1 < *F* ≤ 0.15	288	5.64
0.15 < *F* ≤ 0.2	123	2.41
0.2 < *F* ≤ 0.25	47	0.92
0.25 < *F* ≤ 0.3	26	0.51
0.3 < *F* ≤ 0.35	9	0.18
0.35 < *F* ≤ 0.4	1	0.02

Within this population, 52.66% were not inbred and 47.34% of dogs showed some degree of inbreeding. In total, 90.33% of the study population showed an inbreeding coefficients less than 0.1. The average inbreeding coefficient for the entire study population was 0.0282 and the average inbreeding coefficient in the inbred dogs was 0.0597. The maximum and minimum inbreeding coefficients were 0.3696 and 1.48e-05, respectively.

### Estimation of OSA heritability using a Probit model

**Fig. 2:**
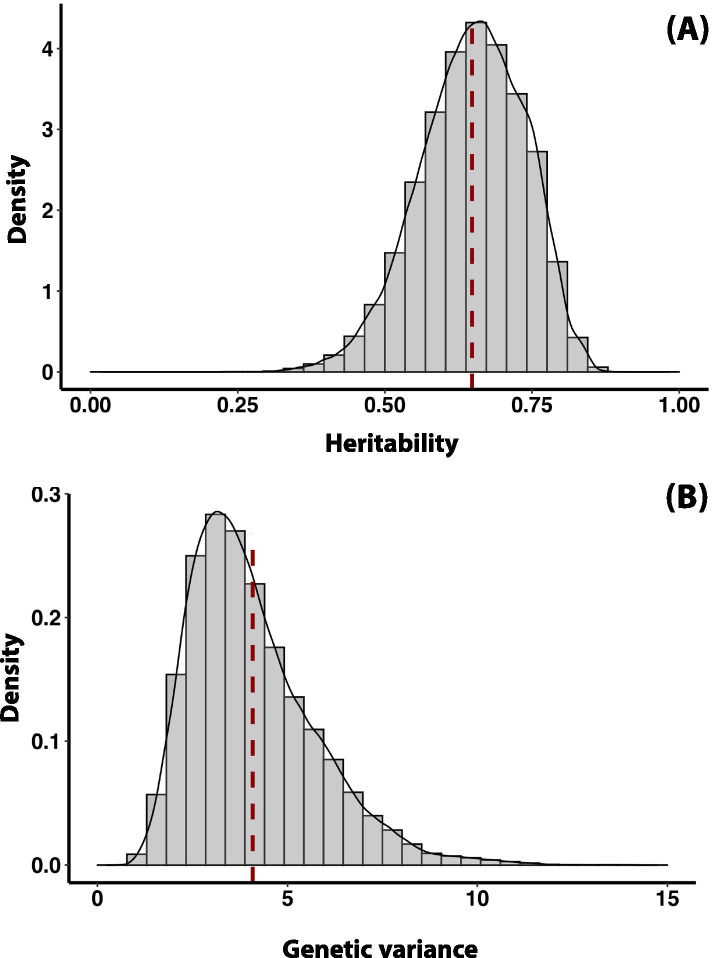
Posterior densities plots for **A** estimated heritability, ${h}_{OSA}^2$ and **B** additive genetic variance, ${\sigma}_a^2$, obtained using probit Bayesian linear mixed model for OSA in Irish Wolfhound. The vertical dashed dark red line indicates the mean of each distribution

To estimate OSA heritability, we used a Probit model. The posterior marginal density and histogram plot of the estimated heritability for OSA is graphically presented (Fig. 2A). The mean of OSA heritability distribution was 0.654 with a standard deviation (SD) of 0.87 (*h*^*2*^_*OSA*_ = 0.648 ± 0.089). The credible interval measured by highest posterior density interval (HPD) at 95% level resulted in lowest and highest interval limits of 0.473 and 0.809, respectively. The quantiles of marginal posterior density of *h*^*2*^_*OSA*_ were 0.461 (2.5%), 0.589 (25%), 0.653 (50%), 0.714 (75%), and 0.801 (97.5%). Also, the posterior mean for the additive genetic variance component (${\sigma}_a^2$), as the numerator of the above heritability equation, was 4.082 ± 1.665. The lower and upper limits of HDP interval for additive genetic variance were 1.450 and 7.4878, respectively (Fig. 2B). The results show that the estimated heritability for OSA follows our hypothesis that OSA is highly heritable. We assumed that estimation of these genetic components using a Bayesian approach along with resemblance between relative’s linkage matrix (**A**) is solely due to additive genetic variation.

### Pedigree disease evaluation

Of the 5110 dogs included in the pedigree analysis, 332 were OSA cases and 360 were assigned as controls. Noteably, there were no OSA affected dogs over 10 years of age found during the construction of this study population. Of the 332 cases, 136 had at least one known parental phenotype. Of these dogs, 68 (50%) had at least one affected parent. Seventeen dogs had known phenotypes of both parents; 5 had 1 affected parent, 4 had 2 affected parents, and 8 had both parents assigned as controls (Table 3).

**Table 3:** Distribution of parent phenotypes of Irish Wolfhounds with and without OSA

Sire phenotype	Dam phenotype	Case	Control	Total
OSA	OSA	4	3	7
OSA	Unknown	35	10	45
Unknown	OSA	24	16	40
OSA	Control	1	4	5
Control	OSA	4	4	8
Unknown	Control	47	47	94
Control	Unknown	15	30	45
Control	Control	8	8	16
Unknown	Unknown	194	238	432
Total		332	360	692

## Discussion

This is the first study to evaluate the heritability of OSA in Irish Wolfhounds. Canine OSA has a poor prognosis, with less than 45% of dogs surviving > 1 year after diagnosis despite treatment [1, 2]. This disease is particularily devastating to the Irish Wolfhound breed, which has been reported to affect between 8 and 20% of the population [4, 5, 19]. OSA is also substantially more common in some breeds than others [19]; diseases that are breed-specific or have substantial breed predisposition have a genetic basis.

The family pedigree and family structure of the dog sample used for this study was also evaluated. The results of this analysis indicated that the pedigree used, consisting of 5110 dogs, was of sufficient size and power to enable an accurate estimation of OSA heritability. It is noteable that 47% of dogs within this population showed some evidence of inbreeding. This is expected, and likely reflective of a study population consisting of a subset of dogs from a pure-bred population. For the Irish Wolfhound breed, this is compounded by the challenge of the breed being less common, narrowing the breeding population further. These challenges are substantial for more unusual or rare breeds of dogs, and are important considerations in investigation of genetic disease as identification of disease associated mutations are important considerations for breed improvement over time. It is also important to note that the inbreeding co-efficients reported in this study relate only to the study population, and cannot be extrapolated to the entire Irish Wolfhound population. Inbreeding leads to loss of diversity at the individual and population levels, which can hinder ability to respond to a changing environment. Having some degree of inbreeding in the closely replated population like in this study is inevitable; inbreeding patterns observed in purebred dogs can be a result from specific breeding practices or founder effects and not be overlay from the current population size [20]. However, the findings in this study are not surprising because smaller populations tend to have proportionally more animals with higher inbreeding coefficients than larger populations.

We estimated the heritability of OSA (*h*^*2*^_*OSA*_) in the Irish Wolfhound to be 0.654, indicating that OSA in the Irish Wolfhound breed is a highly heritable complex disease. This heritability estimate indicates that environmental and epigenetic factors also influence disease risk. These results align with an earlier genome wide association study investigating OSA in the Irish Wolfhound, where only four genomic regions were found to associate with disease [9], as well as a meta-analysis study that indicated in individual dogs, Mendelian-level polygenetic risk can be present [10].

We further investigated our study population by evaluating the distribution of parental phenotypes in Irish Wolfhounds assigned as either a case or control. It is noteable that for dogs that developed OSA, a much higher proportion had at least one parent with an OSA diagnosis when compared to the control population. While this data is challenging to interepret due to a large number of case and control dogs for which at least one parental phenotype was not known, it does support the calculated heritability estimate. A reliable risk calculation based on odds of disease development for animals with a known affected parent is not possible with this current dataset due to the large number of affected offspring with at least one parent not having a reported phenotype.

The results presented also mirror findings of OSA in the closely related Scottish Deerhound, where variance component analysis estimated heritability of OSA to be 0.69; in this study, heritability in the Scottish Deerhound was best modeled using a Mendelian major gene model with dominant expression [12]. The findings from Scottish Deerhound OSA are of particular relevance to the Irish Wolfhound due to breed history. In the mid-nineteenth century, Irish Wolfhounds were nearly extinct as a breed. The breed’s revival was due to a combination of identifying dogs suspected to have descended from older Irish Wolfhound strains and cross breeding with Scottish Deerhounds, Great Dane crosses, and a smattering of other dogs including a Borzoi and a Tibetan Mastiff [21]. This breed history is relevant for two reasons: 1) from a genetic standpoint, current Irish Wolfhounds have substantial relatedness to Scottish Deerhounds, and 2) the Irish Wolfhound population descends from a small subset of dogs, resulting in a genetic bottleneck that likely contributes to high rates of disease prevalence [22].

The pattern and heritability seen in this study is substantially different from the OSA inheritance patterns seen in humans, where OSA is a highly complex disease [7]. Disease traits can have many different modes of inheritance. Simple or Mendelian diseases occur when a single genomic mutation is responsible for disease, which can occur with cancers though what is referred to as “cancer predisposition genes” [23]. This is in contrast to highly complex diseases, where multiple genomic variants spread across the genome, in combination with environmental effects, contribute to disease risk. Most cancers follow a complex disease inheritance pattern. Complex diseases vary in the degree to which genetics and environmental effects influence disease risk, and further vary with regards to degrees of polygenicity [14]. Prior literature has informed us that in dogs OSA is a complex disease but the underlying genetic architecture contributing to disease risk is variable between breeds [9]. In breeds such as the Irish Wolfhound and Scottish Deerhound, where heritability estimates are high and literature suggests a finite number of high impact genetic mutations [10, 11], there is a greater likelihood that identification of a few influential OSA mutations can be identified with further research.

Another important difference between human and canine OSA is that in humans OSA is considered a childhood disease, while OSA in most dog breeds is a disease of aged animals. Noteably, the Irish Wolfhound breed is unusual in this regard, having the youngest age of OSA onset of commonly affected breeds [19].

**Fig. 3:**
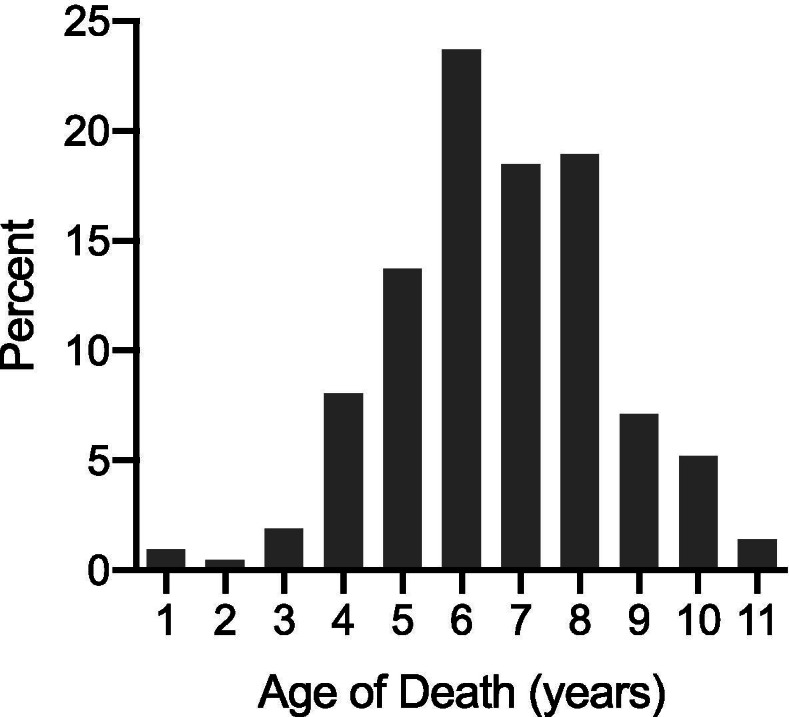
Osteosarcoma Age of Death. In the population used for this preliminary analysis, ninty-3 % osteosarcoma-related deaths of Irish Wolfhounds occur prior to 10 years of age. Please note that this data is based on age of death in OSA-affected Irish Wolfhounds, not age of diagnosis. Data provided by the Irish Wolfhound Foundation, collected between 2000 and 2015. *n* = 211

There are a number of limitations to this study. Data was primarly retrieved through an on-line database of Irish Wolfhound pedigrees, and, therefore, diagnosis of OSA was owner reported; medical records were not able to be obtained. Many dogs in the database were noted to be deceased without a cause of death, and it is likely that a number of animals who were affected by OSA were not assigned a phenotype. The decision to define control dogs as those Irish Wolfhounds over 10 years of age was based on a number of factors, including prior literature [19] and data provided by the Irish Wolfhound Foundation (Fig. 3). The Irish Wolfhound, like many giant breeds, has a shorter lifespan [24], with mean lifespan estimates from the past 50 years varing between 6.5 and 8.8 years [25, 26]. Thus 10 years of age is considered very old for this breed. This control age choice was further supported by a lack of any dogs over 10 years of age being reported to have died of OSA during creation of the heritability matrixes used for this study. However, it is possible that some dogs who lived beyond 10 years of age were affected with OSA but were not reported as having such in the Irish Wolfhound database used. It is also possible that dogs who only lived to 10 years of age may have developed OSA had they lived longer. It is difficult to predict how these factors may have influenced results, but the large sample size was purposely created to increase power and decrease the effect of any inaccuracies in phenotyping that may have occurred.

## Conclusions

The heritability estimate determined through pedigree analysis indicates OSA in the Irish Wolfhound is a complex disease with a substantial genetic contribution. These findings, in addition to prior studies [9–13], indicate the likely presence of a finite number of high-risk genomic variants that contribute to disease risk. Overall, this work strongly supports further investigation into the genetic contribution to OSA in the Irish Wolfhound breed.

## Methods

### Study population

Data for the heritability study were obtained from the Irish Wolfhound Database [16], a publically available domain with entries are provided by owners. All dogs in this database are considered pure-bred Irish Wolfhounds.

### Definition of phenotype

Irish Wolfhounds were considered cases if they were diagnosed with OSA prior to death and this information was recorded within the Irish Wolfhound Database [16]. Dogs were considered controls if they lived to be over 10 years of age and did not have OSA reported as a diagnosis or cause of death. The age cut-off for control dogs was determined through preliminary data involving the evaluation of a cohort of 211 Irish Wolfhounds known to have died from OSA (Fig. 3) and prior literature [19].

### Pedigree analysis and numerator relationship matrix

Using pedigrees available through the Irish Wolfhound Database, we created a population-based pedigree of 5110 pure-bred Irish Wolfhounds. This pedigree was analyzed using the CFC software tool [27] through which we obtained general information on the structure of pedigrees, checked for the pedigree errors and loops, and extracted a list of summary statistics, as presented in Table 1.

To fit a linear mixed model and estimate variance components and subsequently obtain heritability of disease, we created a numerator relationships linkage matrix (**A**), which reflects the additive genetic relationships (variance-covariance) between all animals. This matrix was originally introduced by Henderson [28], to account for covariance between random effects, and to use information from relatives in estimation of breeding values and variance components. We created the **A** matrix in the R environment and obtained inbreeding coefficients for each individual dog. Computationally, the **A** matrix is a square symmetric, and has dimensions equal to the total number of individuals in the pedigree (*N* × *N*). Its off-diagonal elements (*A*_*ik*_), are twice the probability that an allele drawn from individual *i* is identical by descent to an allele in individual *k* and its diagonal element is (*A*_*ii*_ = 1 + *F*_*i*_), where *F*_*i*_ indicates the inbreeding coefficient for animal *i*. The MCMCglmm R package [29] was then used to fit the model.

### Estimation of family-based heritability

We implemented a probit Bayesian linear mixed model to estimate genetic variance components and obtain the posterior distribution of heritability for OSA in Irish Wolfhound dogs. Our disease outcomes followed a binary disease response (i.e., case/control), so the assumption was made that OSA in the Irish Wolfhound is a trait dictated by an underlying normally distributed latent variable. An individual’s value of the liability determines which of two categories a given individual falls into. Therefore, we consider that the probability of OSA for the j-th outcome on the i-th dog (*p*_*ij*_), can be modeled as:

$$logit\ \left({p}_{ij}\right)=\log \left[\frac{p_{ij}}{1-{p}_{ij}}\right]=\mu +{X}_{ij}{\beta}_{ij}+{a}_i+{e}_{ij}$$

Where μ is the total population mean which is constant common to all dogs; *X*_*ij*_ can be dedicated to a set of recorded nongenetic / systematic effects where in this analysis involved only sex effect (1 for male and 2 for female); *β*_*ij*_ is the unknown vector of effects associated with sex effect; *a*_*i*_ is the additive genetic contribution to OSA status for the i-th dog and assumed to be sampled from a multivariate normal density with $\boldsymbol{a}\mid {\sigma}_a^2\sim N\left(0,\boldsymbol{A}{\sigma}_a^2\right)$, so it was a vector of liability values for all 5110 dogs, and ***A*** being the numerator relationship matrix as described above. In this model the residuals were assumed to follow a multivariate normal distribution with mean zero and identity variance (**I**) i.e., $\boldsymbol{e}\sim N\left(0,\mathbf{I}{\sigma}_e^2\right)$. Notably, in a binary or ordinal latent model, the residual variance may not be identifiable [30]. A solution proposed to overcome this complexity is to fix one of the variance components to a known constant C (for example ${\sigma}_e^2$ = C). In this analysis, we fixed ${\sigma}_e^2$ =1, allowing us to re-parameterize our model in terms of heritability. A Bayesian framework, as implemented in the MCMCglmm R package [29], available through the R language [31], was used to fit the model and gauge the related genetic parameters.

The prior distribution for the fixed effects was the default setting that is an independent normal density with null means and variances of e10, which is a relevant and consensual choice. The prior distribution for the random genetic effect and variance components were assumed to follow an inverse-Wishart density, which is a standard assumption. In the MCMCglmm package, this distribution is parametrized by two parameters, nu (*ν*) and V. For our analysis, we set nu = 0.002 and V = 1, to permit a long flat right-skewness distribution, which effectively creates a diffuse prior for the unknown additive genetic variance ${\sigma}_a^2$. Estimates of the posterior density for the unknown parameters were generated through a single Monte Carlo Markov chain. A Gibbs sampler was used to obtain posterior distributions. A burn-in of 50,000 samples followed by an additional 1,000,000 samples, thinned by a factor of five, resulted in 200,000 samples which were used during the MCMC iterations to obtain the marginal posterior distributions of parameters. Then, for each sample, the heritability of OSA was calculated using the following formula:

$${h}_{OSA}^2=\frac{\sigma_a^2}{\sigma_a^2+{\sigma}_e^2+1}$$

## Data Availability

The datasets used for this study are available from the corresponding author on reasonable request.

## Abbreviations

HPD: Highest posterior density interval
OSA: Osteosarcoma
